# Assessing the impact of climate variability on maize yields in the different regions of Ghana—A machine learning perspective

**DOI:** 10.1371/journal.pone.0305762

**Published:** 2024-06-25

**Authors:** Samuel Asante Gyamerah, Clement Asare, Henry Ofoe Agbi-Kaeser, Frank Baffour-Ata

**Affiliations:** 1 Department of Statistics and Actuarial Science, Kwame Nkrumah University of Science and Technology, Kumasi, Ghana; 2 Department of Environmental Science, Kwame Nkrumah University of Science and Technology, Kumasi, Ghana; MRC Unit The Gambia at LSHTM, GAMBIA

## Abstract

Climate variability has become one of the most pressing issues of our time, affecting various aspects of the environment, including the agriculture sector. This study examines the impact of climate variability on Ghana’s maize yield for all agro-ecological zones and administrative regions in Ghana using annual data from 1992 to 2019. The study also employs the stacking ensemble learning model (SELM) in predicting the maize yield in the different regions taking random forest (RF), support vector machine (SVM), gradient boosting (GB), decision tree (DT), and linear regression (LR) as base models. The findings of the study reveal that maize production in the regions of Ghana is inconsistent, with some regions having high variability. All the climate variables considered have positive impact on maize yield, with a lesser variability of temperature in the Guinea savanna zones and a higher temperature variability in the Volta Region. Carbon dioxide (*CO*_2_) also plays a significant role in predicting maize yield across all regions of Ghana. Among the machine learning models utilized, the stacking ensemble model consistently performed better in many regions such as in the Western, Upper East, Upper West, and Greater Accra regions. These findings are important in understanding the impact of climate variability on the yield of maize in Ghana, highlighting regional disparities in maize yield in the country, and highlighting the need for advanced techniques for forecasting, which are important for further investigation and interventions for agricultural planning and decision-making on food security in Ghana.

## Introduction

### Background of the study

The variability of the climate has emerged as a critical concern in our era, impacting different facets of our surroundings, such as the agricultural industry. According to Porter et al. [1], climate variability has a greater impact on agricultural production as compared to other factors due to their interaction. The extent of these effects is determined by different climate variables such as temperature, precipitation, sunshine, humidity, carbon dioxide (*CO*_2_), water vapor, rainfall, and greenhouse gases. Although crop yield can be impacted by climatic variables including rainfall, solar radiation, and wind speed [2, 3], precipitation, temperature, and carbon dioxide (*CO*_2_) are seen as the most influential climatic variables on crop yield [4, 5]. Crop production is hampered by low temperatures [6] and crop yields are significantly affected by high temperatures [7]. Similarly, high precipitation, which leads to flooding, damages crops [8], and low precipitation, which leads to drought, damages crop as well [9]. Therefore, any variations on these climate variables may also have a major impact on the agricultural sector [10].

Agriculture is significant to Ghana’s economy and livelihoods, hence understanding the influence of climate variability on agricultural production is critical. Maize production is one of the most significant agricultural products, ranking third place behind rice and wheat in terms of production of cereal crops in the world [11]. In Ghana, maize is a staple crop and contributes to most of the agricultural production and consumption in the country. According to research, Ghana produced more than 2.9 million metric tons of maize in 2019, the most since 2010, and 3 million metric tons of maize in 2020, continuing a rising trend that has been evident since 2016 [10]. Maize is grown in all the country’s agro-ecological zones, and it is frequently grown by smallholder farmers in rain-fed conditions [12]. As a result, any aggravation of climate-related factors, such as drought, altered precipitation patterns, and heat stress, is accountable for some of the major issues with maize production in Ghana. The results, however, vary by zone. Ghana has six main agro-ecological zones, which are the Guinea savannah zone, Sudan savannah zone, transitional zone, deciduous forest zone, coastal savannah zone, Rain Forest zone [12]. These agro-ecological zones can be grouped into four subgroups [13] based on maize yield in Ghana (i.e., Transitional Zone, Rain Forest Zone, Guinea Savannah Zone, and Coastal Savanna Zone). Temperature and sunshine are likely to have major impact on maize yield in the Guinea savanna zone since they are the hottest zones in the country [14], and precipitation is also likely to have a major impact in the rain forest zone and the coastal savanna zone [10, 15]. Moreover, average temperature is likely to have a major impact in the transitional zone [10].

The impact of climate change on cereal harvests, such as wheat, rice, and maize, has been extensively studied in Asia [16–20] and North America [21–23]. However, there are limited studies in sub-Saharan Africa, especially considering their regional differences, which is of great concern given the region’s high dependence on agriculture for food security and economic development. Given the dependency of maize yield on rainfall in many parts of the African continent, Gachene et al. [24] emphasized the vulnerability of cereal yields in the sub-Saharan regions. They reviewed numerous elements, such as extreme weather events, increased *CO*_2_, weed competition, and insect pests, that may lead to a probable decline in crop production. According to their findings, crop yields in Sub-Saharan Africa (SSA) are projected to decline by more than 10% by 2055. This decline was attributed primarily to the increased occurrence of extreme weather events and the interaction of elevated *CO*_2_ levels with temperature and rainfall. According to the findings from studies in [4, 7, 25], even though temperature is a significant factor influencing maize production, rainfall and water availability are more restrictive elements. Nevertheless, rising temperatures can make some regions crop yield more vulnerable [7]. An interesting study is the one by Acquah and Kyei [26], who discovered in their paper that, an increase in temperature in Ghana is probably going to make maize output variability worse, whereas an increase in rainfall is probably going to address the problem. A study by Adjei and Kyerematen [27] also found that over the previous five years, rainfall in Ghana’s transitional zone (Nkoranza South Municipality) had significantly decreased, which had a detrimental effect on maize agricultural operations. Even though the study by Atiah et al. [25] highlighted that climate factors, such as rainfall, only contribute to a minor portion (about 4.2% of the fluctuations in annual maize output in Ghana). Their findings do indicate that climate factors account for 40.8% of maize yield under drier-than-normal rainfall conditions, indicating that rainfall does have a significant impact on crop productivity in Ghana. The effect of specific climate factors (temperature and rainfall) on maize production in the Ejura-Sekyedumase Municipality, Ghana, was examined by Cudjoe et al. [12] utilizing trend analysis, regression analysis, and chi-square analysis. The research’s conclusions show that there is clear climate variability in the study area and that it significantly affects crop productivity. Another study by Baffour-Ata et al. [28] investigated how climate factors, such as yearly rainfall, its onset and cessation, the number of dry days, and temperature, affect the growth of a few different crops, including millet, sorghum, rice, groundnuts, and maize in northern Ghana. They discovered that number of dry days, temperature, onset, yearly precipitation, and cessation explained about 25% of the variation in maize production using the Mann-Kendall trend test and multiple regression analysis. Evidently, there is a lack of research addressing the impact of climate variables on maize yield across all agro-ecological zones and administrative regions in Ghana. Consequently, it is essential to fill this gap, and this study aims to do so.

Given the necessity of assessing the impact of climate variability on crop yield, various techniques have been developed that enable the analysis of complex interactions between climate variables and crop yield. These techniques involve the use of statistical models, machine learning algorithms, and remote sensing data, among others. Lobell and Burke [29] predicted the possible impacts of climate variabilities on crop yield using three statistical models (time series, panel, and cross-sectional models). They discovered that depending on the climate variable and spatial scale being considered, the performance of statistical models differs. The responses to temperature change were more accurately predicted by panel and cross-sectional models than the responses to precipitation change, which only used data from a single site. The historical period utilized for training was less important for the models based on numerous sites, and performance improved when individual sites were averaged across countries. However, according to Gyamerah et al. [30], it can be difficult to precisely quantify and estimate the impact of climate variability on crop output using statistical techniques because of the complexity and non-linear relationship between crop yield and climate variables. Crane-Droesch [31] utilized a semiparametric deep neural network variant, employed as a machine learning technique to account for the nonlinear interactions in high-dimensional datasets as well as known parametric structure and undiscovered cross-sectional variability. According to their study, the machine learning technique outperforms the classical statistical methods in forecasting the yields of years withheld during model training, according to data on maize yields from the US Midwest, which they used to demonstrate the efficacy of their methodology. In addition, their findings revealed significant adverse impact of climate variability on maize yield, while these effects are less severe than those predicted using conventional statistical techniques. They did this by employing scenarios from a variety of climate models. Appiah-Badu et al. [32] used a methodical and exacting approach to evaluate the effectiveness of several classification algorithms in predicting rainfall in various ecological zones of Ghana. The study employed five well-known machine learning techniques for classification: multilayer perceptron, decision tree, extreme gradient boosting, random forest, and K-nearest neighbor. In their investigation, MLP, XGB, and RF all performed well at the three training-to-testing ratios of 90:10, 80:20, and 70:30 whereas KNN did poorly in all zones. Cedric et al. [33] also suggested a machine learning-based prediction method to forecast the yield of six crops at the national level in West African countries throughout the course of the year. The prediction system was developed using a decision tree, k-nearest neighbor, and multivariate logistic regression models. They combine agricultural yields, chemical data, climate, and weather data in their analysis. According to the study, all three machine learning models produced favorable accuracy, with the decision tree model performing best with 95.3% accuracy. To prevent overfitting during cross-validation, they additionally used a hyper-parameter tuning strategy. The prediction of the K-Nearest Neighbor model and the decision tree model were shown to relate to the expected data when the authors examine the relationship between the predicted results and the expected outcomes, suggesting that the models function as expected. However, using clusters for small datasets may result in overfitting when using forecasting models [34]. To overcome this issue, they used the stacking ensemble learning method (SELM), which helped them increase their generalization capacity. Li et al. [35] also used the Stacking Ensemble Learning framework to integrate three effective machine learning algorithms: support vector machine, random forest, and K-nearest neighbors as base-models. Over the course of 34 years, the study collected daily weather information as well as information on soybean output from 173 county-level administrative districts and meteorological stations in China’s two primary soybean-growing regions. Their findings suggested that the stacking model provides more accuracy and versatility. They also discovered that the stacking prediction model of soybean meteorological yield provides a novel method for successfully predicting soybean production with a MAPE of less than 5%. Based on the findings presented above, the stacking ensemble learning model has demonstrated promising results for reliably predicting maize yield using meteorological data and machine learning techniques. With our data spanning 27 years, the stacking ensemble model can be used to develop a high-precision and reliable model to analyze the impact of climate variabilities on maize yield prediction.

This study considers all the regions in Ghana. This fills a large information void by including all the four ecological zones in Ghana, which encompass all the regions in the country, as opposed to prior studies that only focused on only one region [12, 28, 36], only one zone [27, 37], or selected zones [38, 39]. The study provides a more thorough assessment of the effect of climate change on maize yields in Ghana by considering all ecological zones and all the regions within each zone, making its conclusions reliable and applicable to the entire nation. Even though Atiah et al. [25] found that weather variables significantly influence maize yield variability, the effects of climatic variability on crop production are complex and poorly understood. Machine learning algorithms have the capacity to examine intricate connections between climatic and weather variables and crop productivity. Machine learning algorithms for predicting agricultural output based on numerous meteorological and climatic elements have a better level of accuracy than the current time-consuming statistical procedures since climate variables are nonlinear [33]. Even though machine learning (ML) approaches have a high level of accuracy, their potential applications in agricultural yield prediction have not been realized due to a lack of more reliable methods [40]. By including different model types and architectural characteristics across the models, ensemble learning model forecasts can increase generalization and prediction accuracy [41]. To address the problem of agricultural yield prediction, it is advantageous to introduce an ensemble learning method [35]. Based on the review of related literature, SSA nations, including Ghana, have paucity of ensemble-based ML algorithms in analyzing the impact of climate variability on crop yields. Additionally, due to its superior prediction accuracy, the stacking ensemble model algorithm is suggested in this paper to combine the algorithms of the LR, SVM, DT, RF, and GB to assess the impact of climate variables on maize yield and to fill the previously mentioned study gap.

Our study aims at achieving the following goals: (i) to identify the key climatic variables that have the most significant impact on maize yield in each region; (ii) to assess the impacts of climate variability on the yield of maize in each region of Ghana; (iii) to develop a reliable predictive model using machine learning algorithms that can capture the volatile, non-stationary, and non-linear nature of the climate variables and accurately forecast maize yield under different climatic conditions; (iv) to provide policymakers in Ghana with evidence-based recommendations on how to address the challenges posed by climate change and ensure food security for the country’s growing population; and finally, (v) to add to the growing literature of the predictive power of stacking ensemble machine learning model to agricultural and climate change studies.

### Paper structure

This study is divided into five primary sections. Aside from the study’s background, the paper is arranged in the following manner. The second section outlines the empirical data, the data preprocessing techniques applied, the machine learning models suggested for maize yield predictions, and the methods used for empirical evaluation. After that, the main results of the study are presented in the third section. We then present a discussion of key findings in section four. Finally, we conclude the study and present some recommendations for future studies in section five.

## Materials and methods

### Areas under study

Table 1 shows the districts under each region in Ghana, and the agro-ecological zones are presented in Table 2.

**Table 1:** The regions in Ghana and major districts where there is maize production.

Region	Major Districts
Western	Wassa Amanfi, Aowin-Suaman, Wassa East, Shama Ahanta East, Sefwi-Wiaso, West Ahanta, Mporhor, Wassa West, Bibiani-Anhwiaso-Bekwai, Juabeso-Bea, East Nzema, Jomoro
Central	Awutu-Efutu-Senya, Upper Denkyira, Twifo-Herman/L.Den, Komenda-Edina-Equafo, Assin-Fosu, Gomoa, Mfantsiman, Ajumako-Essiam-Enyana, Cape Coast, Agona, Asikuma-Odoben-Brakwa, Abura-Asebu-Kwamankese
Volta	Kpando, Adidome, Sogakope, Akatsi, Keta, Denu/Dzodze (Ketu), Hohoe, Jasikan, Nkwanta, Kadjebi, Keta-Krachi
Northern	Tonlon/Kumbugu, Gushiegu/Karaga, Damango, Zabzugu/Tatale, Yendi, Bimbila, Walewale, Gambaga, Tamale, Sevelugu/Nanton, Salaga, Bole, Saboba/Chereponi
Ashanti	Adansi-North, Amansie East, Amansie West, Ahafo Ano North, Asante Akim North, Ejura Sekyeredumase, Sekyere West, Kumasi Metropolitan Assembly, Sekyere East, Adansi West, Ahafo Ano South, Atwima, Ejisu Juaben, Bosumtwe Kwanwoma, Kwabre Sekyere, Offinso, Adansi East, Asante Akim South, Afigya Sekyere
Eastern	Kwahu North, Birim South, Fanteakwa, Birim North, West Akim, Akwapim North, Suhum/Kraboa/Coalta, Akwapim South, New Juabeng, East Akim, Kwahu South, Manya Krobo, Yilo Krobo, Kwaebibirim/Kade, Asuogyaman
Greater Accra	Tema, Ga, West Dangbe, East Dangbe
Brong Ahafo	Dorma Ahenkro, Sunyani, Asutifi, Wenchi, Berekum, Tano, Kintampo, Sene, Nkoranza, Jaman, Atebubu, Techiman, Asunafo
Upper West	Wa, Lawra, Tumu, Jirapa, Nadowli
Upper East	Builsa, Kasina/Nankani, Bongo, Bolgatanga, Bawku East, Bawku West

**Table 2:** The agro-ecological zones and their maize cultivation calendar in Ghana.

Agro-Ecological Zone	Administrative	Seasons for cultivation of Maize	Planting period-onset	length of the cropping cycle	Harvesting period-end
	Coverage areas				
Guinea Savannah zone	Northern region, Upper west Region, Upper East region	One season	20-May	90–120 days	10-September
Transitional Zone	Major part of Brong Ahafo, northern part of Ashanti Region, part of Volta region	First season	20-March	90–120 days	20-August
		Second season	20-July	90–120 days	10-December
Rain Forest Zone	Eastern region, southern part of Ashanti, and Western region, parts of Volta, Brong Ahafo, Greater Accra, and Central region	First season	10-March	90–120 days	10-August
		Second season	20-July	90–120 days	20-December
Coastal zone	Parts of Central region, Greater Accra region, Eastern and Volta region	First season	20-March	90–120 days	20-September
		Second season	20-July	90–120 days	20-December

### Data collection

Monthly data for the climate variables (average temperature, minimum temperature, maximum temperature, precipitation, and *CO*_2_) were obtained from the Ghana Meteorological Agency for all the regions in Ghana from 1992 to 2019. The reason for incorporating average, minimum, and maximum temperatures as predictors in the same model stemmed from the intention to encompass a thorough depiction of temperature dynamics and their potential influence on the response variable, motivated by existing studies [10, 25, 42]. For this study, the ten (10) administrative regions that existed between 1992 and 2019 were used. The Statistics, Research, and Information Directorate (SRID) of Ghana’s Ministry of Food and Agriculture provided the yearly average maize yield in metric tons per hectare (MT/ha) for each district from 1992 to 2019. The average maize yield for each district corresponds to the ratio of the district’s estimated production to the district’s estimated cropped area. Motivated by Preety et al. [43], the LINEST function in Excel is used to handle missing values in the original data before aggregating it further. The specific software used for this interpolation task was Excel because of its user-friendly interface and built-in features for linear interpolation. It is worth noting that although Excel was used for interpolation, the subsequent analysis and modeling were carried out using Python with pandas and scikit-learn.

### Data preprocessing

The data from May to September were aggregated to create the annual data for each climatic variable in the major regions in the Guinea Savannah zone since that is the cropping season for maize in the Upper West, Upper East, and Northern parts of Ghana. From Table 2, the Transitional, Rain Forest, and Coastal Savannah zones have two major cropping seasons for maize (March–August/September and July–December); therefore, the data from March to December is aggregated to obtain the annual cropping season climate data. To perform this aggregation, the values of the pertinent variables for each month during the time were added up, and the mean value for the entire period was then determined. As a result, each variable for every year of the study had a single value. In addressing the temporal structure of the data, we conducted an Augmented Dickey-Fuller (ADF) test to evaluate the stationarity of the ’maize yield’ time series. Based on the ADF test, it was determined that the series from all regions exhibited nonstationarity, as indicated by p-values exceeding 0.05. Subsequently, detrending was performed using the ‘detrend’ function from ‘scipy.signal’ library in Python, and the detrended variable was further deseasonalized by subtracting its lagged value, assuming a yearly seasonality. To achieve stationarity, differencing was applied to the deseasonalized data. This comprehensive approach aimed to mitigate temporal patterns, trends, and seasonality in the ’maize yield’ time series, ensuring that the data is suitable for subsequent analysis and modeling using the fixed-effect regression models.

We utilized both the MinMaxScaler and the StandardScaler from the sklearn library in Python to scale the input variables. It became evident that the StandardScaler yielded superior results compared to the MinMaxScaler. Consequently, we opted to standardize the input variables using the standard scaler. Besides, since the climate variables have different units, the standard scaler become the appropriate choice. The decision for standardization was made to ensure that all variables were aligned on a consistent scale, aiming to fit them within a standardized range. Subsequent to making predictions, an inverse scaling procedure was employed to transform the standardized predictions back to their original scales, maintaining the interpretability of the results in the context of the original data units. The datasets were arbitrary divided into two subsets, one comprising the test set (20%) for validating and the other containing the training set (80%) for modeling. The entire analysis was conducted using the Python programming language.

### Model establishment

Assessing the impact of climate variability on crop yield is an important task in agricultural research because it provides useful insights into how climate variability affects food security. The goal of this study is to develop a model for measuring the impact of climate variability on maize yield. To accomplish this, we employed a variety of machine learning algorithms, including multiple linear regression, random forest, support vector machines, decision trees, and gradient boosting. These algorithms were used to construct a single model (the stacking ensemble model) that offers high accuracy in assessing the impact of the climate variables on maize. Previous research [35, 40, 44–47] have shown that ensemble learning can perform better than individual machine learning methods. The machine learning models under consideration are established in this section.

### The base models establishment

*Multiple linear regression*. A supervised learning strategy designed specifically for regression is linear regression. A linear function is used to depict the relationship between the input and output variables. By minimizing the sum of the squared deviations between the expected and actual output values, a straight line is found. In this study, since our independent variables are more than one, we use the multiple linear regression model. The general equation of a multiple linear regression is given in Eq (1).

$$Y_{t}=\beta_{0}+\sum_{i=1}^{n}\beta_{i}\cdot x_{i,t}+\varepsilon_{t}$$ (1)

Where, *Y*_*t*_ is the response variable at time t *x*_*i*,*t*_ is the predictor variable *i* at time t, *β*_*i*_ is the regression coefficients that determines the impact of each climate variables, *β*_0_ is the intercept, and *ε*_*t*_ is the error term at time t. For this studies, Eq (1) can be reformulated as shown in Eq (2)

$$Y_{t}=\beta_{0}+\beta_{1}x_{1,t}+\beta_{2}x_{2,t}+\beta_{3}x_{3,t}+\beta_{4}x_{4,t}+\beta_{5}x_{5,t}+\varepsilon_{t}$$ (2)

where *Y*_*t*_ is the maize yield; *x*_1,*t*_ = average temperature; *x*_2,*t*_ = maximum temperature; *x*_3,*t*_ = minimum temperature; *x*_4,*t*_ = precipitation; *x*_5,*t*_ = *CO*_2_ at time t, *ε*_*t*_ is the error term at time t or residual representing the difference between the predicted value and the actual value of *Y*_*t*_ also known as the unobservable factors affecting *Y*_*t*_; *β*_1_, *β*_2_, *β*_3_, *β*_4_, *β*_5_ captures respectively the effect of *x*_1,*t*_, *x*_2,*t*_, *x*_3,*t*_, *x*_4,*t*_, *x*_5,*t*_ on *Y*_*t*_, and *β*_0_ is the intercept of the equation.

*Decision trees*. A decision tree is a supervised learning method that may be applied to both classification and regression problems. The tree is created by recursively dividing the data according to the values of the input variables. Each leaf node of the tree represents a class label (in the case of classification) or a prediction (in the case of regression), and each internal node of the tree represents a test on an input variable. We formulated the decision tree regression for this model given the dependent variable *Y* as:

$$Y=\sum_{j=1}^{K}C_{j}∙1_{R_{j}}\left(X\right)$$ (3)

Where *Y* is the predicted value which is maize in this case, *K* is the number of terminal nodes or leaves in the decision tree, *C*_*j*_ is the constant value assigned to the *j*_*th*_ leaf node, representing the predicted value of *Y* for that region, $1_{R_{j}}\left(X\right)$ represent an indicator function that takes the value 1 if the output vector *X* falls within the rectangular region *R*_*j*_ and 0 otherwise.

*Random forest*. The ensemble learning method known as a random forest produces numerous decision trees during training and outputs the mean prediction (regression) or mode of the classes (classification) of the individual trees. Each tree in a random forest is trained using a different subset of the characteristics and a different portion of the training data. By averaging all the trees’ projections, the final prediction is produced. In this study, the Random Forest regression was considered. Mathematically, given an input vector *X* (Climate variables), the random forest model predicts the output $\hat{y}$ (maize yield) as:

$$\hat{y}=\frac{1}{T}\sum_{t=1}^{T}f_{t}\left(X\right)+\varepsilon$$ (4)

In this study, Eq 4 can be written as:

$$Y_{t}=\frac{1}{T}\sum_{t=1}^{T}f_{t}\left(x_{1,t},x_{2,t},x_{3,t},x_{4,t}{,x}_{5,t}\right)+\varepsilon_{t}$$ (5)

Where *T* represents the number of decision trees in the ensemble, *f*_*t*_ is the function that maps the independent variables (climate variables) to the dependent variable *Y*(maize yield). *Y*_*t*_ is the maize yield; *x*_1,*t*_ = average temperature; *x*_2,*t*_ = maximum temperature; *x*_3,*t*_ = minimum temperature; *x*_4,*t*_ = precipitation; *x*_5,*t*_ = *CO*_2_, *ε*_*t*_ is the error term or residual that represents the difference between the predicted value and the actual value of the dependent variable and *f*_*t*_(*x*) is the prediction of the *t*_*th*_ tree. In a random forest model, the function is a combination of multiple decision trees that each provide a prediction for the dependent variable.

*Support vector machine*. A support vector machine is a type of supervised learning technique that can be used for classification as well as regression. The SVM finds a hyperplane that optimally separates the input into various groups (in classification) or predicts the output values (in regression). The hyperplane is chosen in such a way that the margin between the different classes is maximized. Mathematically, the hyperplane is defined by the equation:

w∙x+b=0 (6)

Where *w* is the weight vector, *x* is the input vector, and *b* is the bias term. The prediction $\hat{y}$ for a new input vector *x* is given by:

$$\hat{y}=sign\left(w∙x+b\right)$$ (7)

The "*sign*" refers to the sign of the output of the decision tree model. The output of a decision tree is a discrete set of values based on the rules and splits that the tree has learned from the training data. The sign function is used to map these discrete values to either +1 or -1. The positive sign (+1) indicates that the sample is predicted to belong to the positive class (or have a positive target value), while the negative sign (-1) indicates that the sample is predicted to belong to the negative class (or have a negative target value).

*Gradient boosting*. Gradient Boosting is an ensemble technique that combines predictions generated by different weak models to produce a powerful forecasting model. The "gradient" in the term "gradient boosting" refers to the fact that the technique iteratively adds new models to the ensemble to minimize a loss function. Each new model is taught to fix the errors committed by the existing ensemble. The direction and size of the ensemble updates are chosen by the algorithm using gradient descent optimization. Given a training dataset with input features (Climate variables) represented by *X* and corresponding target values (maize yield) represented by *y*, where *X* = {(*x*_*m*_, *y*_*m*_), *for m* = 1, 2, …*n*}, gradient boosting aims to construct an ensemble model say *F*(*x*) that approximates the true target values of *y*. With an initial model *F*_0_(*x*), the training for each iteration *m* = 1 to *M* is represented in Eq 8 which computes the negative gradient of the loss function with respect to the current ensemble model’s prediction. With a weak model say, *w*_*m*_(*x*), the updated ensemble model can be represented in Eq 8, where *w*_*m*_(*x*) approximates the negative gradients and corrects the errors made by the current ensemble model, *ρ*_*m*_ is the optimal learning rate or step size which controls the contribution of the weak model to the ensemble. The final ensemble model in the gradient boosting is shown in Eq 10.


$$\gamma_{m}=-\nabla_{\gamma }L(y,F_{m-1}\left(x\right))$$ (8)

$$F_{m}\left(x\right)=F_{m-1}\left(x\right)+\rho_{m}∙w_{m}(x)$$ (9)

$$F\left(x\right)=F_{0}\left(x\right)+\rho_{1}∙w_{1}\left(x\right)+\rho_{2}∙w_{2}\left(x\right)+\cdots +\rho_{m}∙w_{m}\left(x\right)$$ (10)

#### The stacking ensemble learning model establishment

Stacking is a type of ensemble learning technique that combines multiple base models to improve the overall performance of the model. The predictions from various base models are used as input in the stacking ensemble learning model, which learns how to combine the predictions to make the final prediction through a secondary learning process known as meta-learning, which improves the accuracy and stability of the prediction [48]. The predictive performance of models in the available input subsets is estimated and evaluated in this work using cross-validation techniques. This technique is effective in avoiding overfitting and would be helpful in handling small datasets [34]. Motivated by studies in [40, 47, 49], this study employs a 5-fold cross-validation. This is a well-known cross-validation strategy for optimizing model hyperparameters and evaluating model performance [47]. Each model is trained on ’k-1’ folds of the k-folded input training dataset before being validated on the fold that was not used in training. The technique is applied k times, with the validation fold being changed each time. The forecasts are then stored in train-meta so that they can be utilized as features or inputs for the stacking model.

We defined parameter grids for the base models’ hyperparameter tuning. For the linear regression model, we kept the default values for its parameters, setting "fit intercept" to true and "normalize" to false. In the case of the decision tree model, we specified the options for the maximum depth, which could be none, 5, 10, or 15. The minimum sample split had choices of 2, 5, or 10, and the minimum sample leaf could be 1, 2, or 4. Regarding the random forest model, we provided options for the number of estimators: 100, 200, or 500. We also set the maximum depth to none, 5, 10, or 15, and the minimum sample split and minimum sample leaf had the same choices as the decision tree model. For the support vector machine model, we set the degree parameter to select from 2, 3, or 4. The kernel had options of linear, poly, rbf, or sigmoid. Additionally, the ’C’ parameter, representing the size of the margin value for the support vector regression model to select from either 0.1, 1, or 10. In the gradient boosting model, we defined the learning rate to select from 0.1, 0.05, or 0.01, and the number of estimators could be 100, 200, or 500. To fine-tune each base model’s hyperparameters, we utilized GridSearchCV with 5-fold cross-validation and evaluated them using the negative mean squared error scoring parameter. GridSearchCV is an approach in machine learning that focuses on tuning hyperparameters to identify the most effective combination for a given model systematically. This method necessitates the definition of a parameter grid, which outlines the potential values for each hyperparameter to be explored. In our investigation, the selection of the parameter grid is influenced by the recommendations outlined in [35, 42, 47]. We also employed the linear kernel support vector regression method as the meta regressor motivated by Panigrahi et al. [50].

**Fig 1:**
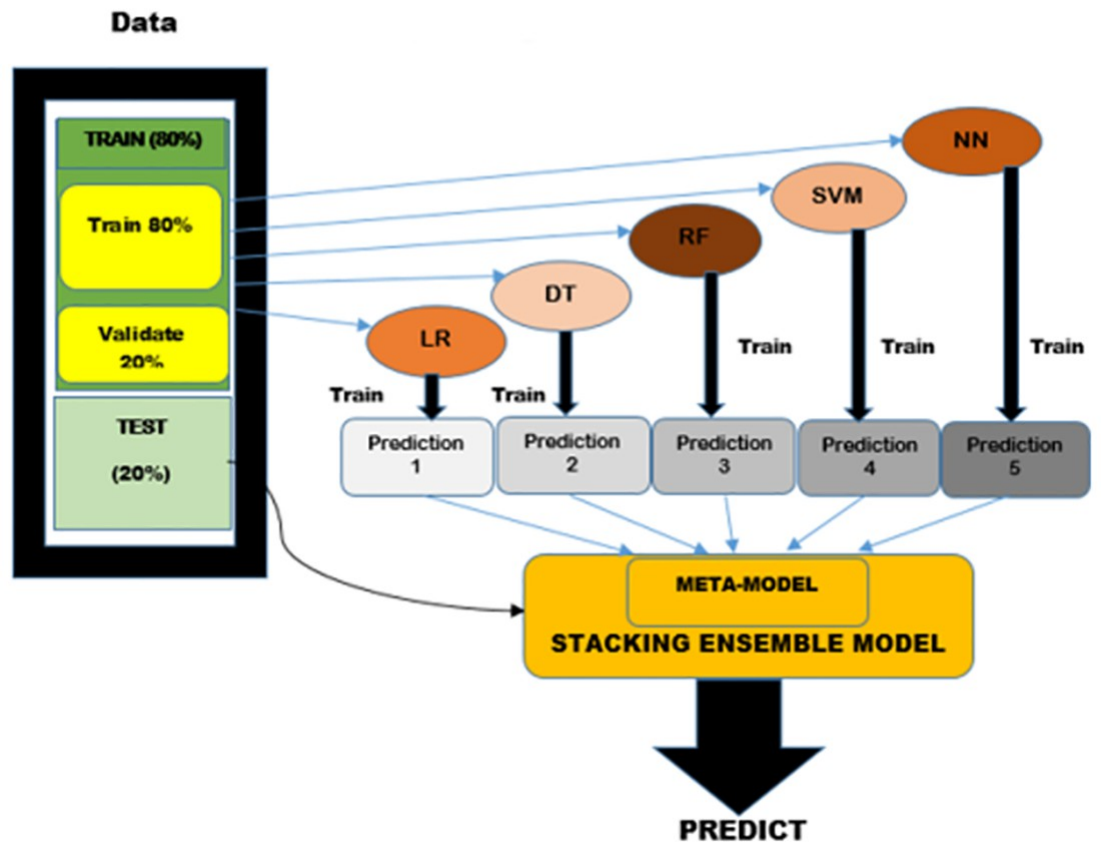
The model framework of stacking ensemble learning model.

The basic idea behind stacking is to use the strengths of multiple base models to compensate for their weaknesses. Each base model is trained on the training data using a different algorithm and generates predictions for the validation or test data. The predictions from the base models are then combined using a meta-model, which is trained on the predictions generated by the base models. The meta-model learns how to weigh the contributions of the base models based on their past performance, and generates the final prediction as shown in Fig 1.

#### The evaluation metrics of the model performances

In this study, statistical measures were utilized as metrics to assess the performance of the proposed stacking ensemble prediction model. To evaluate the model’s ability to fit the data, performance evaluation metrics were employed. The proposed stacking ensemble model and the base models were evaluated based on the mean square error (MSE) and the mean absolute error (MAE). The formulas for MSE and MAE can be found in Eqs 11 and 12, respectively. Hence, the preference is for smaller values as they signify improved model performance in minimizing the discrepancies between predicted and actual values.


$$MSE=\frac{\sum_{i=1}^{n}{\left(y_{i}-{\hat{y}}_{i}\right)}^{2}}{n}$$ (11)

$$MAE=\frac{\sum_{i=1}^{n}\left|y_{i}-{\hat{y}}_{i}\right|}{n}$$ (12)

### Empirical evaluation

The descriptive statistics of maize yield are shown in Table 3. From the Table, the yield of maize ranged from 0.33 MT/Ha in 1992 in the Upper West Region to a maximum of 5.57 mt/ha in 2019 in the Greater Accra Region. The average yield of maize varied across all the regions. The highest (2.01 MT/Ha) and lowest (1.17 mt/ha) average maize yields were detected in the Eastern and Greater-Accra regions, respectively. This indicates that the production of maize was more stable in the Eastern Region than in the Greater Accra Region. To compare the variability of maize yield in all the Regions in Ghana, the coefficient of variation (CVar) is employed. From the coefficient of variation as shown in Table 3, Around the mean maize yield of 1.17 mt/ha, the Greater Accra region has the largest coefficient of variation (CVar), at 77%. This implies that the yield of maize in this region is subject to significant variability over time. In comparison to other regions, the yield of maize in the Greater Accra region exhibits a more annual uneven performance. The Western region, on the other hand, exhibits the lowest coefficient of variation (CVar), which is 8% around the mean yield of 1.40 mt/ha. This suggests that compared to other regions, maize output in the Western Region is more consistent and has lesser variability over time. This means that a more dependable and predictable production pattern is shown by the constancy of maize yield in this region.

**Table 3:** Descriptive statistics of the maize yield for each of the regions in Ghana.

Region	Count	Min	Max	Mean	CVar (%)
Ashanti	28.00	1.13	3.02	1.57	26.0
Northern	28.00	0.60	1.97	1.34	26.0
Eastern	28.00	1.36	2.98	2.01	22.0
Volta	28.00	0.99	2.36	1.65	18.0
Central	28.00	0.80	2.82	1.73	30.0
Upper East	28.00	0.50	2.98	1.36	37.0
Upper West	28.00	0.33	2.69	1.53	29.0
Brong Ahafo	28.00	1.41	2.45	1.88	12.0
Greater Accra	28.00	0.62	5.57	1.17	77.0
Western	28.00	1.03	1.55	1.40	8.0

Although there is a lot of variability in the Greater Accra region, it is vital to remember that its mean yield (1.17 mt/ha) is lower than that of all the regions in Ghana (see Table 3). This indicates that, on average, the Greater Accra region produces less maize than the other nine (9) regions. Low average yield and high variability indicates that there may be difficulties or variables affecting maize cultivation in the Greater Accra region that cause both low average yield and high yield variability. Therefore, future research should analyze the variables affecting maize yield in this region (Greater Accra Region).

#### Analysis of the climate variables

Climate variables are essential for understanding and appreciating the dynamics of climate patterns and environmental conditions in different regions. Analyses of climate variables such as average temperature, maximum temperature, minimum temperature, precipitation, and *CO*_2_ levels, provide vital insights into the changes and trends that influence Ghana’s maize yield. We can quantify the central tendency and relative variability of these climate variables using key statistical measures such as the coefficient of variation and mean. The extent of fluctuations in temperature, precipitation, and carbon dioxide levels is analyzed by looking at the coefficient of variation and mean as shown in Table 4. Table 4 shows the mean and coefficient of variation for each climate variable across Ghana’s regions.

**Table 4:** Mean and coefficient of variation analysis of the climate variables.

Regions	Measures	Climate Variables				
		Avg Temp	Min Temp	Max Temp	Precip	CO_2_
Ashanti	mean	26.98	22.34	31.67	1316.83	0.39
	CVar (%)	0.99	1.18	0.96	10.33	35.06
Volta	mean	27.42	22.67	32.22	1238.68	0.39
	CVar (%)	**1.19**	**1.57**	0.99	12.17	35.06
Eastern	mean	27.01	22.39	31.67	1236.59	0.39
	CVar (%)	1.08	1.34	0.99	12.34	35.06
Northern	mean	28.28	22.69	33.92	1133.44	0.39
	CVar (%)	1.01	1.31	0.92	8.52	35.06
Upper East	mean	**28.95**	23.02	**34.94**	997.57	0.39
	CVar (%)	1.03	1.41	0.92	8.92	35.06
Upper West	mean	28.61	22.92	34.35	1010.48	0.39
	CVar (%)	1.04	1.29	**1.00**	9.04	35.06
Central	mean	26.82	22.98	30.7	1230.02	0.39
	CVar (%)	1.00	1.15	0.97	11.93	35.06
Western	mean	26.92	22.89	31.01	**1491.63**	0.39
	CVar (%)	0.92	1.07	0.92	11.39	35.06
Brong Ahafo	mean	27.39	22.45	32.36	1209.11	0.39
	CVar (%)	0.98	1.20	0.94	8.53	35.06
Greater Accra	mean	27.34	**23.50**	31.23	950.9	0.39
	CVar (%)	1.08	1.35	0.98	**13.17**	35.06

Note: The bold values represent the highest and precip represents precipitation.

According to the analysis in Table 4, with a mean temperature of 27.42°C, the Volta region has the highest coefficient of variation (CVar) of 1.19%, while the Upper East region has the highest mean temperature (28.95°C) of all the regions with a coefficient of variation of 1.03%. This signifies that the temperature in the Volta region experiences more significant fluctuations over time compared to other regions. The Upper East region, on the other hand, has the highest mean temperature, indicating that it experiences greater temperature on average than the other regions. However, the coefficient of variation in the Upper East region (1.03%) is lower than in the Volta region (1.19%), indicating that the temperature in the Upper East region is less variable than in the Volta region. In comparison to the other regions, the Northern, Upper East, and Upper West regions are noted to have higher average temperatures. This is since these areas are in the Guinea Savannah Zone, which is regarded as the warmest region in the nation and does not have frequent rainfall.

With a mean precipitation of 950.9 mm, the Greater Accra region has the highest coefficient of variation (CV) of 13.17%, whereas the Western region has the highest mean precipitation of all regions with 1491.63 and a coefficient of variation of 11.39%. That is, the precipitation in the Greater Accra region is more volatile than in the other regions. This means that precipitation in the Greater Accra region varies more significantly over time. The Western region, on the other hand, has the greatest mean precipitation of all the regions, suggesting that it receives more rainfall on average than the other regions. However, the coefficient of variation in the Western region (11.39%) is lower than in the Greater Accra region (13.17%), indicating that precipitation in the Western region is less variable than in the Greater Accra region. The average CO_2_ level across all regions is 0.39, with a coefficient of variation (CV) of 35.06%. The mean value of 0.39 represents the average carbon dioxide concentration throughout the regions under consideration. Carbon dioxide is a greenhouse gas that contributes significantly to climate change. This mean number indicates that the regions have a given level of carbon dioxide in the atmosphere on average. The coefficient of variation of 35.06% shows the regional variability in carbon dioxide levels. The comparatively high coefficient of variation of 35.06% suggests that carbon dioxide concentrations in Ghana’s various regions vary significantly over time.

## Results

### Trends of maize yield for each region in Ghana

**Fig 2:**
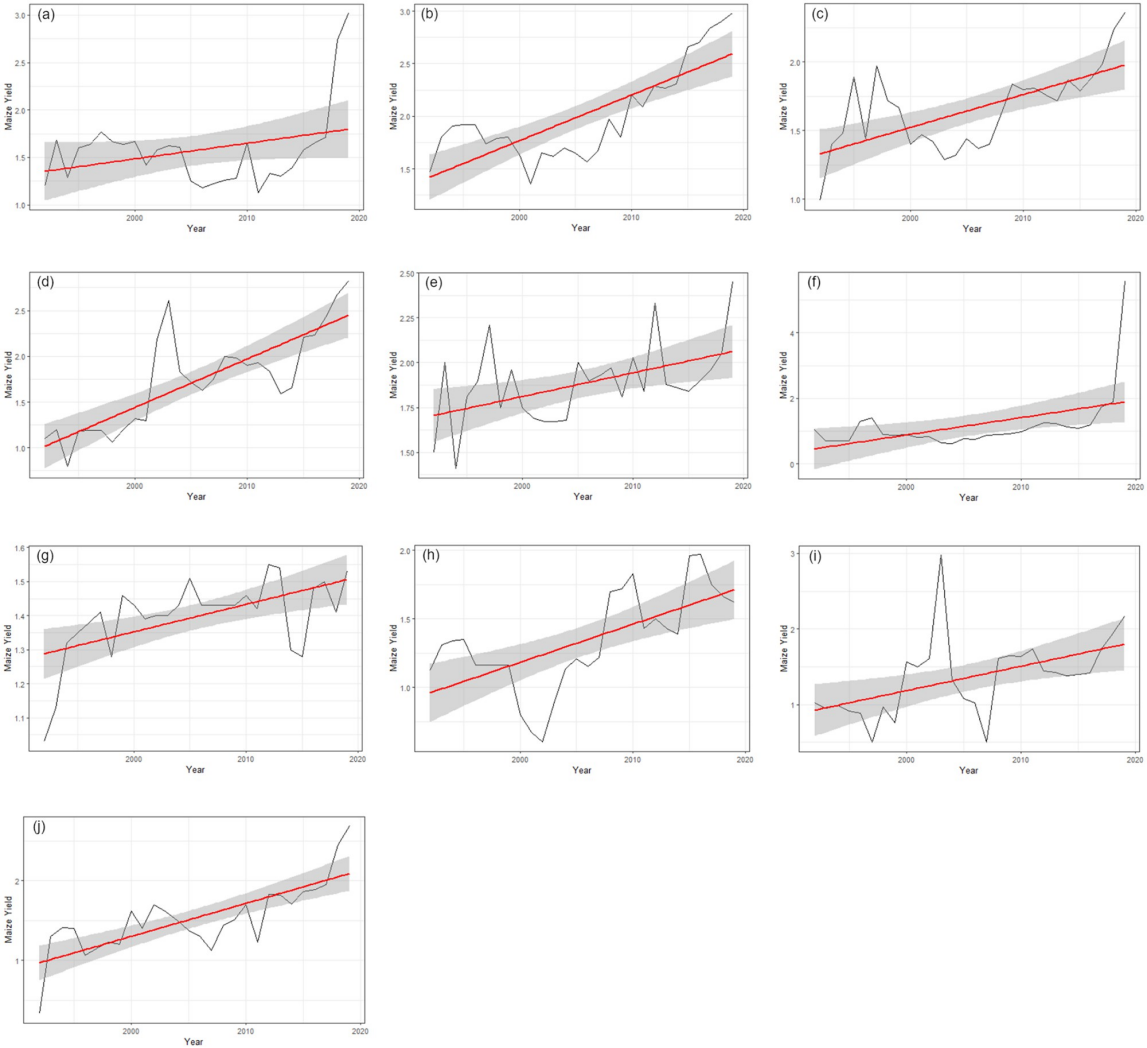
Trend of maize yield in each of the regions of Ghana.

In this section, we investigate the variation and trends in maize yield for each region over the period under study in Ghana. Fig 2 clearly show that the yield of maize has increased significantly across all the regions in Ghana. In Fig 2, the maximum maize yield in all the regions we observed in 2019 except for the Upper East and the Western region which recorded the maximum maize yield in 2003 and 2012 respectively. However, it is observed that it started rising after the year 2015. This increasing trend of maize yield in the regions can be attributed to several factors which are discussed subsequently.

### Impact of climate variables on maize yield

**Fig 3:**
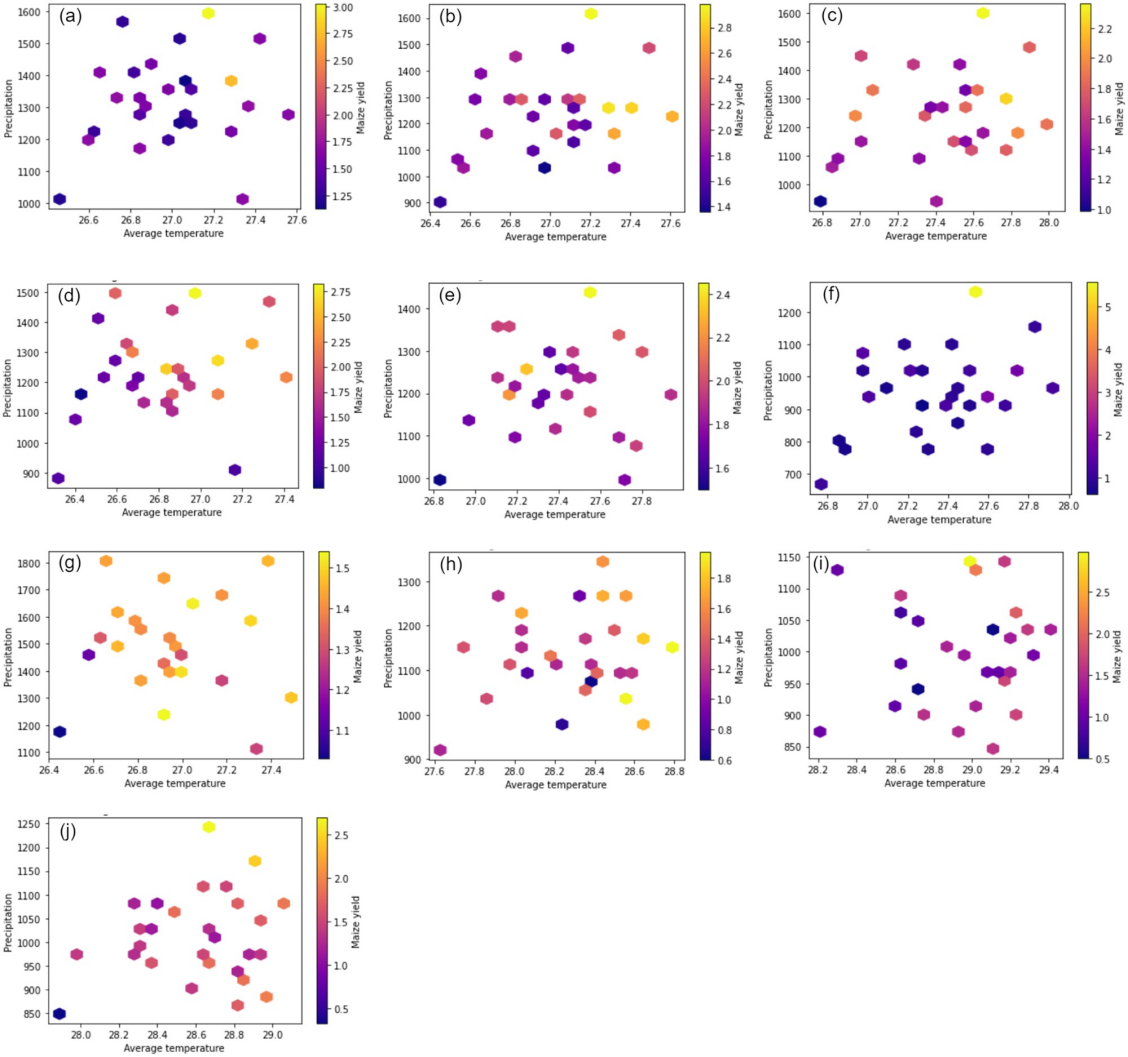
Combined impact of average temperature and precipitation on maize yield in the different regions of Ghana.

Although there are numerous climate factors that affect maize output, temperature and precipitation have the greatest effects on crop productivity [4]. Fig 3 shows how average temperature and precipitation affect maize yield in each region. We fixed the threshold for maximum maize yield (see Fig 3) at 2.0 mt/ha for all the regions apart from the Northern and Western regions, where the thresholds for maximum maize yield were set at 1.8 mt/ha and 1.5 mt/ha, respectively. This is because the maximum yields of maize in either of these two regions never exceeded 2.0 mt/ha.

In the Ashanti region of Ghana, the maize yield is influenced by specific temperature and precipitation conditions. Based on the data presented in Fig 3a, it is observed that higher maize yields are obtained when the average temperature ranges between 27.10°C and 27.30°C, and the precipitation amounts fall between 1388.80 mm and 1593.53 mm. The optimal combination is found at an average temperature of 27.15°C and a precipitation total of 1593.53 mm, which leads to the maximum maize yield. In the Central region as shown in Fig 3d, it is evident that higher maize yields are obtained when the average temperature ranges between 26.60°C and 27.50°C, and the precipitation amounts fall between 1217 mm and 1495.00 mm. The optimal combination is found at an average temperature of 26.98°C and a precipitation total of 1489.66 mm, which leads to the maximum maize yield. In the Eastern region as depicted in Fig 3b, the production of higher maize yields is associated with average temperatures between 26.86°C and 27.61°C and precipitation amounts ranging from 1168.53 mm to 1615.79 mm. The maximum maize yield occurs at an average temperature of 27.19°C and a precipitation total of 1615.79 mm. In Volta region as shown in Fig 3c, it is observed that higher maize yields are obtained when the average temperature ranges between 27.64°C and 27.76°C, and the precipitation amounts fall between 1314.30 mm and 1600.11 mm. The optimal combination is found at an average temperature of 27.64°C and a precipitation total of 1600.11 mm, which leads to the maximum maize yield. In the Brong Ahafo region as shown in Fig 3e, higher maize yields are obtained when average temperatures range from 27.16°C to 27.81°C and precipitation amounts fall between 1204.32 mm and 1437.83 mm. The maximum maize yield occurs at an average temperature of 27.53°C and a precipitation total of 1437.83 mm. In the Greater Accra region (Fig 3f), a maximum maize yield is produced with an average temperature of 27.53°C and a precipitation total of 1262.70 mm. This region clearly benefits from higher precipitation, as it is a significant factor for achieving higher yields. In the Upper East region (Fig 3i), maize yield increases with average temperatures between 28.98°C and 29.03°C and precipitation amounts ranging from 1124.53 mm to 1142.28 mm. The optimal maize yield is observed when the average temperature is 28.98°C, and the precipitation measures 1142.28 mm. Similarly, in the Upper West region (Fig 3j), maize yield increases with average temperatures ranging from 28.68°C to 28.92°C and precipitation between 1168.38 mm and 1242.41 mm. The optimal maize yield in the region is achieved at an average temperature of 28.68°C and a precipitation total of 1242.41 mm. In the Northern region (Fig 3h), a maximum maize yield is produced with average temperatures ranging from 28.58°C to 28.79°C and precipitation ranging from 1030.30 mm to 1166.60 mm. The data in Fig 3h clearly illustrates the beneficial effect of higher average temperatures on maize yield in this region. In the Western region (Fig 3g), maximum maize yields are obtained when average temperatures range from 26.72°C to 27.33°C and precipitation amounts fall between 1223.53 mm and 1663.42 mm. The optimal combination occurs at an average temperature of 26.92°C and a precipitation total of 1223.53 mm, resulting in the maximum maize yield.

Based on the findings from Fig 3, it can be concluded that increased precipitation is beneficial for maize yield in Ghana. Additionally, the optimal range of average temperatures for maize yield varies depending on the region, ranging from 26.00°C to 28.00°C. Conversely, lower average temperatures and less precipitation do not contribute to the production of a good maize yield, as evidenced by the data presented in these figures.

### Machine learning models implementation

Using 5-fold cross-validation and Grid Search, the results of the stacking ensemble learning model, which includes the optimal parameters of the base models (Gradient Boosting, Linear Regression, Decision Tree, Support Vector Machine, and Random Forest), are shown in Table 5. Table 5 provides a thorough summary of these ideal parameter values, showing the precise setups that produced the best results when used with the stacking ensemble building. Utilizing the unique capabilities of each base model, the stacking ensemble learning employs the prediction of the optimal outcomes of each base model to build the final prediction. Each region’s unique ideal parameters were achieved (see Table 5), and the optimal parameter for each region was identified. This will be useful in predicting maize yield based on climate variables.

**Table 5:** The results of the optimal parameters for each base model after performing Grid Search.

Regions	Optimal parameters values of each base model after Grid Search with 5-fold CV											
	DT			RF				SVM			GB	
	max depth	min sample_split	min_sample_leaf	n_estimators	max depth	min sample_split	min_sample_leaf	C	Kernel	Degree	n_estimators	Learning rate
Ashanti	5	10	4	100	15	5	4	1	poly	3	100	0.01
Eastern	5	10	4	200	5	10	1	1	rbf	2	100	0.01
Volta	5	2	4	100	15	10	2	1	sigmoid	2	100	0.01
Central	5	10	4	500	5	10	1	1	linear	2	100	0.01
Brong Ahafo	15	2	1	100	5	2	1	0.1	poly	3	100	0.01
Greater Accra	5	5	2	100	10	10	1	0.1	poly	2	100	0.01
Western	5	10	4	200	5	10	4	1	rbf	2	100	0.01
Northern	5	2	4	100	10	10	1	10	poly	2	100	0.01
Upper east	5	2	1	200	10	10	4	1	sigmoid	2	100	0.01
Upper west	15	2	2	100	15	10	4	10	poly	4	100	0.01

### Results of the performance metrics for the models

The statistics in Table 6 demonstrate that, in terms of MSE and the MAE, the LR and the SVM models perform much worse than other single models. As a result, the nonlinear relationship between the climate factors and maize production makes it difficult to predict using either the linear regression model or the support vector machine. The performance of various machine learning models in predicting maize yield across different regions demonstrates notable variations. By evaluating the MSE and the MAE, it is evident that the linear regression model demonstrates excellent performance in the Ashanti region. Additionally, the decision tree outperforms other models in the Eastern region. In the Central region, the random forest model emerges as the most effective. Meanwhile, the support vector machine model shows effectiveness in the Northern and Brong Ahafo regions. The gradient boosting model also stands out as the optimal choice in the Volta and Central regions.

**Table 6:** Performance metric of machine learning models.

REGIONS	METRIC	MACHINE LEARNING MODELS					
		LR	DT	RF	SVM	GB	STACKING
ASHANTI	*MSE*	**0.208**	0.258	0.254	0.219	0.241	0.300
	*MAE*	**0.328**	0.376	0.377	0.336	0.357	0.497
EASTERN	*MSE*	0.051	**0.041**	0.049	0.053	0.044	0.052
	*MAE*	0.148	**0.133**	0.145	0.155	0.135	0.143
VOLTA	*MSE*	0.148	0.170	0.152	0.145	**0.135**	0.047
	*MAE*	0.258	0.280	0.262	0.272	**0.238**	0.281
WESTERN	*MSE*	0.028	0.028	0.027	0.026	0.027	**0.025**
	*MAE*	0.116	0.118	0.112	0.111	0.115	**0.109**
CENTRAL	*MSE*	0.244	0.214	0.153	0.178	**0.148**	0.200
	*MAE*	0.381	0.374	**0.307**	0.350	0.311	0.401
NORTHERN	*MSE*	0.085	0.088	0.083	**0.082**	0.085	0.083
	*MAE*	0.220	0.226	0.222	**0.220**	0.225	0.224
UPPER WEST	*MSE*	0.160	0.278	0.143	0.136	0.157	**0.133**
	*MAE*	0.332	0.434	0.299	0.279	0.328	**0.270**
UPPER EAST	*MSE*	2.259	2.915	1.268	0.953	1.178	**0.866**
	*MAE*	1.080	1.328	0.818	0.725	0.865	**0.725**
BRONG-AHAFO	*MSE*	0.209	0.282	0.211	**0.165**	0.232	0.215
	*MAE*	0.441	0.515	0.443	**0.359**	0.449	0.410
GREATER ACCRA	*MSE*	0.397	0.079	0.090	0.077	0.085	**0.065**
	*MAE*	0.500	0.225	0.246	0.245	0.246	**0.244**

Note: The numbers in bold indicate the best performing model in the particular region.

The Stacking ensemble model also emerged as a top performer in regions many regions like Western, Upper East, Upper West, and Greater Accra, achieving the lowest MSE and MAE values. It is essential to highlight that the regions, particularly Greater Accra, Upper East, and Upper West, exhibit relatively high coefficients of variation, as shown in Table 3. This observation emphasizes the stacking ensemble model’s capability to effectively manage data instances characterized by significant variability in these regions. However, due to the nature of the stacking ensemble learning model, where the deficiencies of underperforming models are compensated by the strengths of other models, the MSE of some base models tended to be slightly lower compared to the MSE of the stacking ensemble learning model in some regions. This emphasizes the importance of careful consideration when choosing the base models for the stacking ensemble approach. As indicated by the findings in Table 6, it becomes apparent that the stacking ensemble learning model excels in predicting maize yield based on diverse climatic variables across various regions in Ghana. Our findings corroborate the impactful performance of the Stacking ensemble model as documented in the study by LI et al. [35]. Moreover, our observations underscore its proficiency in effectively handling small datasets [34], reaffirming its practical utility in diverse scenarios. The stacking ensemble learning model has also proved its effectiveness in handling nonlinear data as suggested by Wang et al. [51].

### Feature importance of the climate variables in predicting maize yield

**Fig 4:**
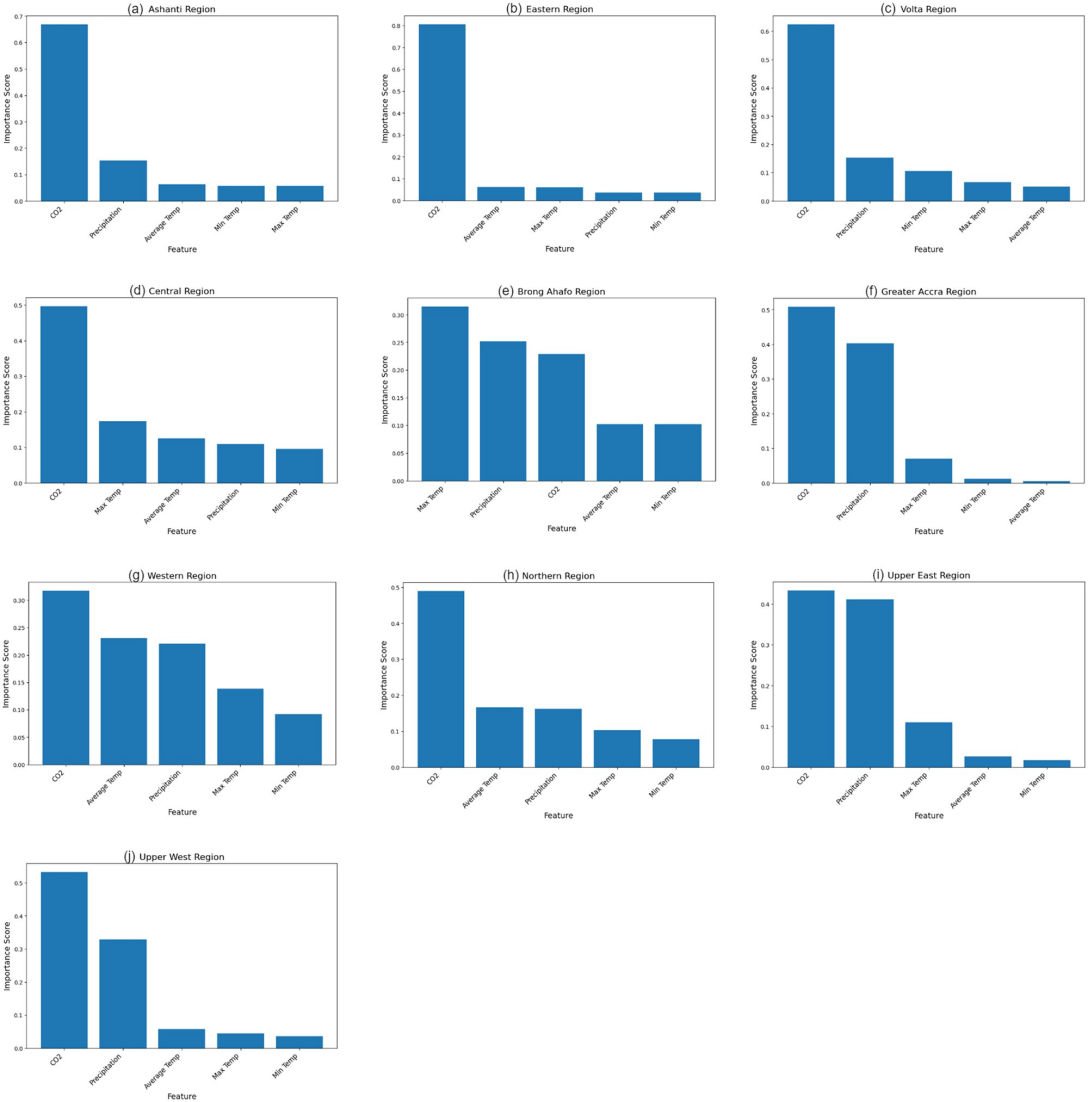
The feature importance of the climate variables in predicting maize yield in each region of Ghana.

Maize yield in Ghana have been experiencing an upward trend as shown in Fig 2. This increasing trend can be attributed to the favorable climatic conditions and optimal policies in maize cultivation. Fig 4 illustrates the feature importance of climate variables in forecasting maize yield. Since random forest performs better across all regions, we base our feature importance calculations for the climatic variables on the random forest Regressor. The statistics below show that *CO*_2_ is a key factor in predicting maize output throughout all of Ghana’s regions, having a strong positive impact on maize yield. This supports the findings of Baffour-Ata et al. [28] which found that the benefit of maize yield across the climate variables is attributed to the positive impact of *CO*_2_. All the climate variables are crucial and have a positive impact on maize yield with none showing negative importance. However, some of them have a stronger impact on maize yield in the regions. The Greater Accra region, the Brong Ahafo region, and the Upper east region all consider precipitation to be a crucial factor in predicting maize yield. Minimum temperature has less impact in the regions that are part of the Guinea savanna zone (Upper West region, Upper East region, and Northern region) and the Rain Forest zone (Ashanti region, Eastern region), but has a strong impact on maize yield in the transitional zone, as evidenced by the prominent position of the feature in the Brong Ahafo region. Additionally, the Brong Ahafo and western areas’ temperatures are more significant compared with those in the rest of the nation.

The upward trend in maize yield can also be attributed to the advancements in agricultural technology and practices. Farmers have gained access to better-quality seeds, improved irrigation systems, and modern farming machinery, all of which have contributed to higher maize yields. Additionally, the government’s focus on agricultural development and support programs has had a positive impact. Through initiatives such as subsidized fertilizer distribution, extension services, and training programs, farmers have been empowered with knowledge and resources to enhance their farming techniques, leading to increased yields. However, the maize yield in the northern region of Ghana (Fig 2h) has been experiencing a downward trend since 2016, therefore, future research should look at the cause of the declining maize yield in the region. The rise in maize yield across all regions in Ghana is not only significant from an agricultural standpoint but also holds economic and food security implications. Higher yields contribute to increased food production, reducing dependency on imports, and ensuring food self-sufficiency. Moreover, surplus maize production can be sold in markets, generating income for farmers, and stimulating economic growth at both local and national levels.

## Discussion

### Summary of Key findings

The agricultural sector of Ghana is heavily dependent on maize production, which also has a significant impact on both economic growth and food security. However, variabilities in maize output across the nation’s regions can pose complications for farmers and decision-makers. We go over the main findings and implications of our assessments on maize yield, climate variables, and their importance in Ghana.

The Greater Accra region shows a high degree of fluctuation in maize production when assessed, which suggests a lack of consistency over time. The Western region, on the other hand, has the lowest coefficient of variation, indicating maize output that is more dependable and predictable. Notably, the Greater Accra region also has a lower average yield in comparison to other regions, suggesting possible factors affecting maize farming there. Further investigation is required to determine and address the variables affecting maize production in the Greater Accra region considering this study. Even though there are variabilities in maize yield in all regions, all of Ghana’s regions have seen a substantial increase in maize output, according to our data. The development of agricultural technology, better farming methods, and government support programs are all responsible for this growing tendency. Productivity has significantly increased because of improved irrigation systems [28], access of high-quality seeds, and sophisticated equipment. Furthermore, measures like subsidized fertilizer delivery, extension services, and training courses have helped farmers become more independent and improved their farming practices [39]. The increase in maize output has implications for the economy and food security, lowering reliance on imports and promoting regional and national economic growth.

Among the climate variables, precipitation patterns, and temperature have significant impact on the productivity of maize. The higher average temperatures are brought on by the Guinea Savannah Zone’s connection to the Upper West, Upper East, and Northern areas. The average temperature in these regions was higher than the average for the rest of the regions, revealing its position as the warmest zone in the nation with less precipitation. The variability in temperature in this region is small indicating how the temperature in the regions vary less over time. The region with the highest variability is the Volta region which experiences significant variations in temperatures. In all regions of Ghana, carbon dioxide (*CO*_2_) also plays a significant role in predicting maize production. In some areas, additional climate factors like precipitation and minimum temperature are also quite crucial. For instance, the Brong Ahafo, Greater Accra, and Upper East regions place a significant emphasis on precipitation. When compared to the Guinea savanna and Rainforest zones, the relevance of lowest temperature is more obvious in the transitional zone (such as Brong Ahafo).

### Policy implications

Our research focuses on maize yield variability in all the regions in Ghana and its contributing components (climate variables), with the employment of machine learning algorithms. We employed machine learning algorithms because of the abilities in handling nonlinear data. Policymakers should enhance agricultural technologies, farming techniques, and assistance programs to maintain maize production. They should also be aware of the factors influencing maize output in Greater Accra and investigate climate-resilient farming methods. As maize yields increase across the country, these initiatives will also help to mitigate Ghana’s agriculture sector’s low average yield and significant yield variability. Overproduction and market sales that arise from this produce economic opportunities and food self-sufficiency, but it’s important to take sustainability and climate change into account. Although greater production is a good thing, it is crucial to prioritize sustainable agriculture and the reduction of greenhouse gases, ensuring that climate-smart agriculture practices reduce the sector’s carbon footprint and boost climate resilience. The information highlights the need to modify agricultural practices to local weather patterns to improve crop output and highlights the complexity of connections between climate and maize yield. Since Ghanaian maize yield assessments provide information on regional variability, yield determinants, and climate variables, this emphasizes the value of localized agriculture that considers regional weather variations and crop growth. The findings of these analyses show the importance of implementing targeted programs and regulations to address local issues, giving Ghana the chance to boost maize production, food security, economic development, and environmental sustainability.

This study’s conclusions are based on analysis and facts based on our data span. To further understand Ghanaian maize yield patterns, new data and studies may require additional analysis. This study lays the groundwork for evidence-based decision-making and measures to boost maize production and agricultural resilience in the country employing the stacking ensemble learning model as an optimal predictive model.

## Conclusion

This study sought to assess the impact of climate variability on all the regions in Ghana’s maize yield and to develop an accurate prediction model by employing five machine learning techniques (SVM, GB, RF, DT and LR) to establish a high-precision prediction model using the Stacking Ensemble Learning model. Our model is optimized using hyperparametric optimization and validated using 5-fold cross-validation to improve its generalizability. We found that *CO*_2_ is a crucial factor in predicting maize yield in all regions of Ghana. Precipitation is important for maize yield in the Greater Accra, Brong Ahafo, and Upper East regions. Minimum temperature is less significant in the Guinea savanna and Rain Forest zones shows a greater impact in the transitional zone, particularly in the Brong Ahafo region. The Stacking model showed higher accuracy and robustness compared to SVM and linear regression models, as confirmed by MSE and MAE evaluation metrics. Stacking outperformed other models in the western and Upper East regions, as well as in the Ashanti region, except for slightly higher MSE in the Eastern region. The Stacking model provides a reliable and accurate approach for predicting maize yield based on climate variables, especially with limited data.

There exist certain constraints within this investigation that pave the way for future exploration. Primarily, the data at our disposal is constrained, potentially impacting the model’s performance. Consequently, future research endeavors could leverage extensive datasets to enhance the accuracy of machine learning models in this field. Moreover, specific machine learning models, such as Linear Regression, are susceptible to multicollinearity. Given that the temperature variables (minimum temperature, maximum temperature, average temperature) may contribute to multicollinearity, alternative regression models should be considered. For instance, one might explore penalized linear regression models like Ridge (L2 regularization), Lasso (L1 regularization), and/or Elastic Net (a combination of L1 and L2 penalties).

## Abbreviations

*CO*_2_: Carbon dioxide
°C: Degree Celsius
DT: Decision Tree
GB: Gradient Boosting
KNN: K-Nearest Neighbor
LR: Linear Regression
MAE: Mean Absolute Error
MLP: Multi-Layer Perceptron
mm: millimeters
MSE: Mean Squared Error
mt/ha: Metric tons per hectare
rbf: Radial Basis function
RF: Random Forest
SELM: Stacking Ensemble Learning Model
SSA: Sub-Saharan Africa
SVM: Support Vector Machine
XGB: XGBoost
